# Desmoplastic Melanoma Arising after 1,064 nm q-Switched Nd:YAG Laser of a Suspected Solar Lentigo

**DOI:** 10.1155/2019/3907671

**Published:** 2019-04-07

**Authors:** Leah Cohen, Sonali Nanda, Martin Zaiac

**Affiliations:** ^1^Department of Dermatology, Florida International University Herbert Wertheim College of Medicine, Miami, FL, USA; ^2^Department of Dermatology and Cutaneous Surgery, University of Miami Miller School of Medicine, Miami, FL, USA; ^3^Chairman of the Department of Dermatology, Florida International University Herbert Wertheim College of Medicine, Miami, FL, USA

## Abstract

**Objectives:**

To present a case of desmoplastic melanoma (DM) arising after laser therapy of a suspected solar lentigo with the 1,064 nm Q-switched (QS) Neodymium:Yttrium-Aluminum-Garnet (Nd:YAG) laser and discuss the safety of treating suspected solar lentigines with laser therapy.

**Methods:**

Case presentation with discussion.

**Results:**

We describe a patient who developed DM after 1,064 nm QS Nd:YAG laser therapy to a suspected solar lentigo.

**Conclusions:**

Limited generalizable studies regarding the safety of laser therapy for solar lentigines exist, specifically for the 1,064 nm QS Nd:YAG laser. Therefore, we recommend caution is taken when considering laser therapy for these lesions, as well as strong consideration for histologic confirmation prior to therapy.

## 1. Introduction

Solar lentigines, or “age spots,” are a common benign cosmetic complaint, present in 90% of the Caucasian population over 60 years old [1]. Removal of these lesions is one of the most frequently performed cosmetic procedures in laser centers around the United States [2]. The use of many Q-switched (QS) lasers including 532 nm Neodymium:Yttrium-Aluminum-Garnet (Nd:YAG) lasers, ruby lasers, and alexandrite lasers are an option for treatment of solar lentigines [3]. While the 1,064 nm QS Nd:YAG laser has demonstrated efficacy in treating melasma and nevus of Ota, to our knowledge no studies have addressed its efficacy for solar lentigines [3]. We report a case of a desmoplastic melanoma (DM) arising after 1,064-nm QS Nd:YAG laser therapy for a suspected solar lentigo of the inferior eyelid. This case introduces an important discussion regarding the safety of treating suspected solar lentigines with laser therapy.

## 2. Case Report

A 72-year-old woman presented with a two-year history of a light brown pigmented lesion located on the lateral segment of her right inferior eyelid. She had no history of nevi, rashes, or scaling of the area. The patient had a past medical history significant for a basal cell carcinoma, melasma, and numerous solar lentigines of the face and neck. She admitted to significant sun exposure and tanning in the past but denied any family history of skin cancer. Three months earlier, she had received laser therapy to the face and neck for skin rejuvenation, using the fractional resurfacing laser at a wavelength of 1,550 nm. Her only reaction to laser therapy was slight erythema and mild swelling. Otherwise, she healed well.

**Figure 1:**
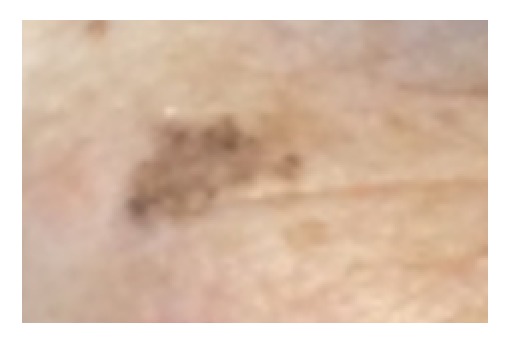
Initially the lesion resembled many other lesions in the patient's sun-exposed skin areas. The lesion was a 3 mm x 2 mm macule colored tan to dark brown involving the lateral segment of the right inferior eyelid.

On examination, the lesion was a flat, well-circumscribed macule, measuring 3 mm x 2 mm, colored tan to dark brown involving the lateral segment of the right inferior eyelid (Figure 1). It appeared similar to many other lentigines on the patient's sun-exposed areas and was clinically correlated to be a solar lentigo. Due to the low clinical suspicion for malignant lesions and sensitive area, biopsy was not obtained. The patient sought cosmetic treatment of the right inferior eyelid lesion and the 1,064 nm QS Nd:YAG laser was used, pulse durations were not recorded.

**Figure 2:**
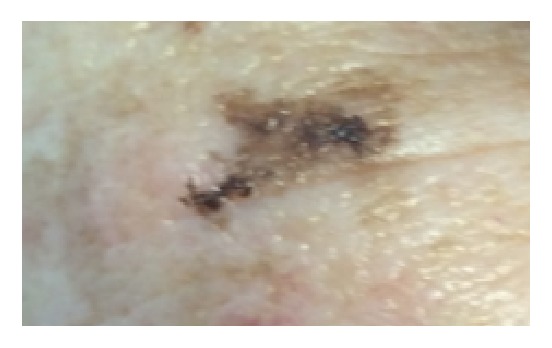
Presentation of lesion after 1,064 nm QS Nd:YAG laser therapy: 4 mm x 2 mm asymmetric macule colored tan to dark brown to black involving the lateral segment of the right inferior eyelid.

Three months after targeted laser treatment of the right inferior eyelid patch, the patient returned complaining of recurrence of the lesion, which appeared to have grown to be a 4 mm x 2 mm asymmetric macule colored tan to dark brown to black (Figure 2). A shave biopsy was taken and returned positive for atypical lentiginous and nested melanocytic proliferation with severe atypia, extending to the lateral margin. The lesion was subsequently excised and final pathology was reported as a desmoplastic melanoma, Clark's level IV, Breslow's thickness 2.5 mm with negative margins. Subsequent follow-up appointments at 2 months, 3 months, 6 months, and 8 months were all negative for clinical recurrence.

## 3. Discussion

We have described a patient who, after receiving 1,064 nm QS Nd:YAG laser treatment of a suspected solar lentigo, developed DM. This case produces several important clinical questions. Was the pigmented lesion at initial presentation a true solar lentigo? If it was a solar lentigo, could it have undergone transformation after laser treatment? Is the 1,064 nm QS Nd:YAG laser an appropriate choice for suspected solar lentigines?

Our current understanding is that there is significant improvement of the appearance of solar lentigines with low-fluence QS 1,064 nm Nd:YAG laser therapy, with many patients experiencing clearance without significant unwanted pigmentary changes or other side effects [3, 4]. A retrospective review of twelve patients with multiple solar lentigines treated with the 1,064 nm QS Nd:YAG laser found that 58% of patients reached near total improvement while 25% reached moderate improvement, with no side effects reported [3]. Despite promising results, this review is one of the only published assessments of the 1,064 nm QS Nd:YAG laser for solar lentigines and is limited by small sample size, retrospective analysis, and lack of histologic confirmation at diagnosis. As our applications of laser therapies expand, it is important to document their side effects to characterize their safety profiles.

When considering the possibility of misdiagnosis, we must consider the broad differential diagnosis of solar lentigines, which includes early seborrheic keratoses, pigmented actinic keratoses, and benign melanocytic nevi, as well as lentigo maligna (LM), a type of melanoma in situ [2]. As solar lentigines are a clinical diagnosis with a wide differential, whose definition may vary between physicians, it is logical that a variety of cosmetic treatments may be performed on these lesions [5]. Several reports have documented LM arising after laser therapies to a pigmented lesion [2, 5, 6]. In a retrospective review of biopsy-proven LM, Hibler et al. concluded that 7.4% of patients with LM had prior cosmetic therapy, 29.7% of which had received laser therapy of some type [5]. The authors also describe eight patients who had a history of benign biopsy prior to their eventual diagnosis of LM, suggesting a possible sampling error during biopsy [5].

Just as LM after laser therapy has been documented, several case series describe invasive melanoma after laser therapy. Zisper et al. describes twelve patients who were treated with ablative lasers, four of which developed nodular malignant melanoma, four developed LM, and four developed other subtypes [7]. Only four of the twelve cases were biopsied prior to laser therapy. When histology was reevaluated after the diagnosis of melanoma, two of the four cases had evidence for pathological misdiagnosis [7]. Most recently, Delker et al. identified eleven patients who developed melanoma after laser therapy; however, specific lasers used were not mentioned [8]. Of the eleven cases, three were LM, one was melanoma in situ, two were superficial spreading, two were nodular, and one was an unknown subtype; 82% had no histologic assessment performed on lesions prior to laser therapy. To our knowledge, there is only one case report documenting a case of DM after laser treatment [9]. Lee et al. describe the development of DM one to two years after treatment with a Nd:YAG laser of an unknown type.

## 4. Conclusion

The diagnosis of melanoma after laser treatment of a pigmented lesion in the same area is not conclusive evidence that a misdiagnosis occurred [2], nor is it conclusive of malignant transformation or, if performed, a misrepresentative biopsy. These three possibilities must be understood so that we can better characterize similar occurrences in the future. By establishing the outcomes associated with laser therapies, physicians and patients can better understand the risk of laser treatment of pigmented lesions. Two issues arise at the heart of this case, the first being that the solar lentigo is a clinical diagnosis, for which the differential diagnosis includes both benign and malignant pigmented entities, and the second is that few evaluations of the 1,064 nm QS Nd:YAG laser for solar lentigines exist. Therefore, we recommend using laser therapies for the treatment of clinically diagnosed solar lentigines with caution. Physicians should consider performing a biopsy of pigmented lesions prior to laser therapy, and if lesions recur after laser therapy, the possibility of malignancy should be considered and followed by immediate biopsy.

## Abbreviations

QS Nd:YAG: Q-switched Neodymium:Yttrium-Aluminum-Garnet
DM: Desmoplastic melanoma
LM: Lentigo maligna.
